# Separation of Glycyrrhizic Acid and Its Derivants from Hydrolyzation in Subcritical Water by Macroporous Resin

**DOI:** 10.3390/molecules25184305

**Published:** 2020-09-19

**Authors:** Rui Fan

**Affiliations:** 1 Department of Nutrition and Food Hygiene, School of Public Health, Peking University, Beijing 100191, China; fanruirf@bjmu.edu.cn; Tel.: +86-18810515491; 2 Beijing Key Laboratory of Functional Food from Plant Resources, College of Food Science and Nutritional Engineering, China Agricultural University, Beijing 100083, China

**Keywords:** glycyrrhizic acid, glycyrrhetinic acid 3-*O*-mono-β-d-glucuronide, glycyrrhetinic acid, adsorption, desorption, resin

## Abstract

Glycyrrhizic acid (GL) and its derivants, glycyrrhetinic acid 3-*O*-mono-β-d-glucuronide (GAMG) and glycyrrhetinic acid (GA) hydrolyzed in subcritical water, are bioactive substances and edulcorators. In this work, a separation strategy for these three substances was established. The effects of adsorbent and eluent were investigated by static/dynamic adsorption and multi-stage desorption with the mechanism analysis. The adsorption of them onto EXA50 resin was well fitted by the pseudo second-order kinetic model. The optimal dynamic adsorption flow rate was 6 bed volume (BV)/h, and water of pH = 12 was used to elute GL at 4 BV/h, then *n*-buthanol was used subsequently to elute GA at 1 BV/h, and finally 90% ethanol was applied to elute GAMG at 2 BV/h. As a result, purities of these compounds increased, which demonstrated that this adsorption-desorption technology was simple and efficient, and indicated the potential for large-scale purification and preparation of GL and its derivants in the future.

## 1. Introduction

Glycyrrhizic acid (GL), the major component in liquorice, has been widely applied in the food and pharmaceutical industries—as it is a natural edulcorator and bioactive compound. However, GL is not in an optimal molecular form to be absorbed in the human intestine, as it may disturb the ionic equilibrium in our body, and thus lead to hypertension [1]. Glycyrrhetinic acid (GA), the aglycon of GL, is the main component responsible for the nutraceutical effects of liquorice [2]. GL and GA exhibit certain similar physiological effects, while the dose of GA to obtain an equal effect is only 2.5% that of GL. Compared with GL, GA is preferred as it is less toxic and is used to design more efficacious agents for cancer chemotherapy [3]. Glycyrrhetinic acid 3-*O*-mono-β-d-glucuronide (GAMG) is formed after cleaving the distal glycosidic bond of GL. As an edulcorator, the sweetness intensity of GAMG is five times greater than that of GL, and GAMG possesses a lower caloric value and, thus, is much safer [4]. GAMG and GL show similar efficacy in the pharmaceutical field including antiviral, anti-inflammatory, anti-ulcer, antiallergic, anti-dote, anti-oxidant, anti-tumor etc., [5,6]; however, GAMG shows a higher biological availability [7]. Therefore, GAMG is considered as an excellent food edulcorator and bioactive component.

GA and GAMG were commonly produced by conventional chemical and biotransformation methods [4,8]. To overcome the drawbacks of the current methods, such as a long hydrolysis time, a complicated operation process, and high production cost, the subcritical water system was introduced to generate the derivants of GL in our previous work [9]. But, the separation technology of GL and its derivants was scarcely reported. If they could be efficiently separated and purified, this could provide great economic and environmental benefits.

Common methods used to separate the single GL, including high speed counter current chromatography, resin adsorption, membrane separation, and molecularly imprinted polymers [10], have been quite limited regarding industrial applications due to low recovery, solvent residue, and being time-consuming. Among these methods, resin was preferred as the more effective selection for adsorption and recovery of constituents because of its different affinity [11]. GL, GAMG, and GA (Figure S1 in the Supplementary Materials) show different molecular polarities because their structures are distinguished by one glucuronic acid, and therefore, their adsorption separation by macroporous resin is feasible. However, no literature is currently available for the separation GL and its hydrolysis products. Therefore, the aim of this study is to develop an efficient process using macroporous resin to separate and purify GL and its derivants from the hydrolysate in subcritical water. I analyzed the characteristics of the static and dynamic adsorption and desorption. The results provide the potential for developing a preparative method for GL and its derivants.

## 2. Results and Discussion

### 2.1. Static Adsorption

#### 2.1.1. Adsorption Kinetics

The initial concentration of adsorbate was a critical parameter in the adsorption process. The influence of the GA initial concentration and dose of EXA50 resin on the adsorption efficiency was evaluated in the present study (see Figure S2 and subchapters 1S and 2S in the Supplementary Materials). The GA initial concentration was 0.8 mg/mL and the dosage of 0.2 g EXA50 resin was selected.

**Figure 1:**
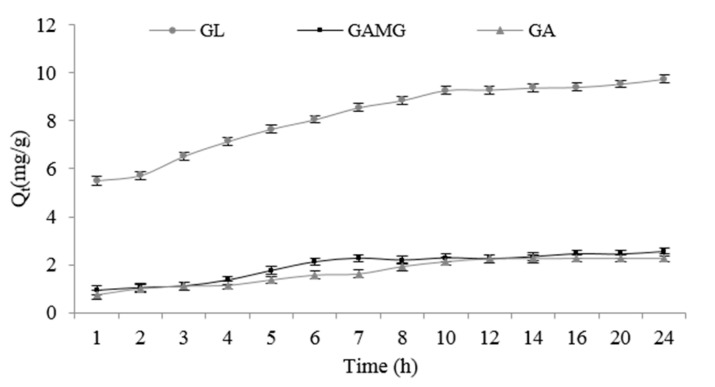
The effect of phase contact time on the adsorption of glycyrrhizic acid (GL), glycyrrhetinic acid 3-*O*-mono-β-d-glucuronide (GAMG), and glycyrrhetinic acid (GA) on the EXA50 resin. Q_t_ (mg/g), the adsorption capacities at *t* time.

The effects of the phase contact time on the adsorption of GL, GAMG, and GA on the EXA50 resin (kinetic curves) are presented in Figure 1. The results showed that the GL adsorption rate of GL was fast initially, with over 70% of the equilibrium capacity reached in the first 5 h, and then the rate changed slowly, and finally reached equilibrium at 9 h. The initial fast GL adsorption was likely ascribed to the accessible mesopores of the rein. The later slower adsorption was indicative of processes with a high intra-particle mass transfer resistance [12]. While, the previous report found that the adsorption equilibrium of GL on Indion 810 could be achieved in 360 min. The obtained equilibrium time was different from the current finding, which might be attributed to the different reins [13]. The adsorption processes of GAMG and GA went through three-stage changes and finally reached the equilibrium after 10 h and 12 h, respectively. Stage 1 had a slight increase step, and the adsorption capacity of GAMG and GA increased slightly in the first 4 h while the adsorption capacity of GL showed a linear rapid increase. This phenomenon indicated that there existed competitive adsorption among GL, GAMG, and GA. Stage 2 quickly increased the step, the adsorption capacity of GAMG and GA increased obviously and then reached the adsorption equilibrium at 10 h and 12 h, respectively. In Stage 3, the diffusion step, the adsorbate molecules gradually diffused into the micropore zone and reached the final equilibrium [14].

**Figure 2:**
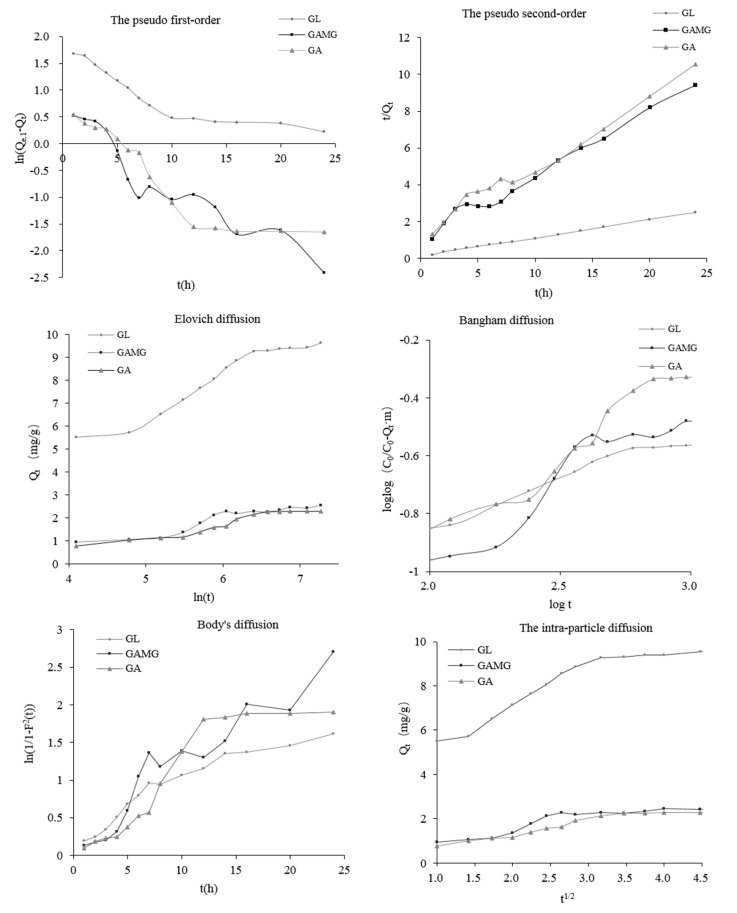
The adsorption kinetics model of GL, GAMG, and GA on the EXA50 resin. *Q_e,_*_1_ (mg/g), the adsorption amount of GL (or GAMG, GA) at equilibrium; *Q_t_* (mg/g), the adsorption amount of GL (or GAMG, GA) at any time *t*(h), *C*_0_ (mg/L), the adsorbate initial concentration; *m* (g/L), the resin mass; F, the fractional attainment of equilibrium at time *t*.

The different kinetics models including the pseudo first-order and pseudo second-order kinetics, the Intra-particle diffusion model, Boyd’s diffusivity model, the Elonvich model, and the Bangham model were utilized to describe the adsorption process. The pseudo-first (PFO) and pseudo-second order (PSO) kinetic models were as described above and their plots are shown in Figure 2. The adsorption capacity and the *k* values (*k*_1_ and *k*_2_ represent the adsorption rate constants of the pseudo-first order and the pseudo-second order model) are listed in Table 1. Compared with the pseudo-first order model, the theoretical *Q_e,cal_* values were coincident with the experimental *Q_e,exp_* ones, the pseudo-second order model was better than the pseudo-first order model. The calculated correlation coefficients (*R*^2^) also indicated that the adsorption rate of GL, GAMG, and GA followed the pseudo second-order equation well. The estimated accuracy (∆*Q* and A*f*) are presented in Table 1, which confirmed that the adsorption of GL, GAMG, and GA followed the pseudo-second-order kinetic model. In addition, the initial adsorption rate V_0_ (GL of 6.977 mg/g·h, GAMG of 0.937 mg/g·h and GA of 0.708 mg/g·h) indicated that initial adsorption rate of the three adsorbates followed the order of V_0_ (GL) > V_0_ (GAMG) >V_0_ (GA), which agreed with the time required for reaching the adsorption equilibrium. As shown in Table 1, the pseudo-second-order equation (*R*^2^ > 0.98) was the most suitable, which revealed that the adsorption process was possibly controlled by two or more rate-limiting steps, such as external diffusion, boundary layer diffusion, and intra-particle diffusion [15]. The findings agreed with the previous report, which was focused on glycyrrhizic acid adsorption using S-8 macroporous resin [16].

Bangham’s equation was used to check whether pore diffusion was the only controlling step during the adsorption process. A desired linear fit in the double logarithmic plot was not observed from Figure 2, which indicated that the diffusion of the adsorbate into the pores of the adsorbents was not the only rate controlling step [17]. Both film and pore-diffusion may also play an important role during adsorption. The similar phenomenon was reported from Barkakat [18].

One of the most useful models for describing such “activated” chemisorptions was the Elovich equation [19]. The plot of adsorbate concentration versus ln*t* for the adsorption process is shown in Figure 2 and the parameters are provided in Table 1. The result indicated that the nature of adsorption was chemisorption and adsorbent surface was energetically heterogeneous [20]. Similarly, the kinetic model of removing congo red dye from wastewater was described as a good correlation with the Elovich model [19].

The kinetic data were also evaluated based on the intra-particle diffusion kinetic model (Weber’s and Morris kinetic diffusion model). It is clearly observed in Figure 2 that the data points could be connected by a straight line that does not pass through the origin, which indicates there were two or more rate-controlling factors of the adsorption rate, including boundary layer diffusion and intraparticle diffusion, in the sorption process [21].

In general, the adsorption of three compounds occurred through four consecutive steps: step 1, bulk diffusion of the adsorbate transferred from the bulk solution to the resin surface; step 2, film diffusion of the adsorbate diffused across the external film surrounding the resin bead; step 3, intra-particle diffusion of the adsorbate migrated into the rein pores; and step 4, the interaction of the adsorbate with available chelating sites on the interior surface of the pores, which was a fast and non-limiting step during adsorption. Therefore, the adsorption rate could be dependent on the film diffusion and/or intra-particle diffusion [22].

From Figure 2, GL’s linear portion (rapid adsorption) was macropore or inter-particle diffusion or boundary layer diffusion, and then the gradual adsorption phase represented the micropore or intra-particle diffusion [18]. Table 1 shows the values of the effective diffusion coefficient, D_e_, which showed a high value in GAMG. Boyd’s diffusion model also indicated that the GAMG adsorption process was less affected by intra-particles diffusion in the adsorption process. Therefore, I suggest that different diffusion models might play an important role in the adsorption kinetics. As can be found from Table 1, the Bangham model was successful in explaining the adsorption data for GA, the intra-particle diffusion models were very suitable in interpreting the adsorption data for GL, and Boyd’s model was reasonable in elucidating the absorption data for GAMG. These phenomena might be attributed to the properties of different adsorbates.

#### 2.1.2. Adsorption Isotherms

(1) The single component (GA) adsorption isotherm

In order to illustrate the adsorption isotherms of GL, GAMG, and GA clearly, the single GA solution was assumed first to describe the equilibrium process. In this investigation, various isotherm models were applied to describe the equilibrium characteristics. The results are shown in Table 2 and Figure S3 in the Supplementary Materials.

The Freundlich isotherm was used to describe the multilayer adsorption of adsorbate onto the heterogeneous surfaces. *K_F_* and *n* indicate the relative adsorption capacity and the adsorption intensity, respectively [23]. In the present work, the values of *n* were all greater than 1, which suggests that the adsorption of GL and its derivants onto EXA50 resin was favorable [24].

The Langmuir isotherm model, widely applied to many monolayer solid-liquid adsorption processes, describes monolayer adsorption on homogeneous surfaces without interaction between adjacent adsorbed molecules [23,25]. Seen from Table 2, the calculated linear regression correlation coefficients indicated that the experimental data were well fitted to the Langmuir model [26]. The values of *R_L_* were all in the range of 0 < *R_L_* < 1; hence, it was a favorable process of the three compounds adsorbing onto the EXA50 resin [23].

The current results showed that both the Langmuir and Freundlich models could be utilized to fit the equilibrium data. Previous studies also reported that they applied these two models to describe the adsorption equilibrium data. Examples included the adsorption of polyphenol from kiwifruit juice [27], the separation and purification of amygdalin [28], where both models showed the satisfactory fitting. The coefficient (*R*^2^) and root mean square error (RMES) indicated that the Freundlich isotherm model fitted the experimental data better, compared to the Langmuir model, implying that the Freundlich model might be relatively more suitable to predict the adsorption of GA on EXA50 resin. Asimilar finding was glycyrrhizic acid on the XDA-1 macroporous resins [29], while there were other reports that glycyrrhizic acid on Indion 810 and S-8 macroporous resin were more suited for the Langmuir isotherm [13,16].

The Tempkin isotherm model considers the chemisorptions of the adsorbate onto the adsorbent. The correlation coefficients (*R*^2^) was less than 0.85, which implied the dissatisfactory representation of the Temkin equation for the adsorption model. A similar conclusion was also reported in another investigation [27].

Bering proposed the isotherm to estimate the mean free energy of the adsorption [30]. The adsorption of GA fit well with the D-R isotherm with a regression coefficient value of 0. The result of single component isotherm model indicated that the experimental data fit well with the Langmuir, Freundlich, and D-R isotherm models. However, the adsorption of GA was best represented by the Freundlich model, which indicated the adsorption onto heterogeneous surface occurred via a physicochemical process involving the -OH and -COOH groups of the resin [19].

(2) The three-component adsorption isotherms

Adsorption in the three-component adsorption was complicated because surface interactions on the resin were involved. Chemical and physical differences between the adsorbate molecules in three-component system often promoted the competition among them [31]. The model parameters and the correlation coefficient *R*^2^ and RMSE for four models are summarized in Table 3. The larger correlation coefficient for the Termin equation suggested that the Termin isotherm model was more suitable for describing the adsorption isotherms of GA. This result was distinct from what was observed with the single component model, likely because of the competition between the three compounds. As found in Figure 2, the adsorption capacity of GA in the three-component system was much lower than that in the single component adsorption, whereas the adsorption capacities of GL and GAMG were higher than that of GA. As the adsorption sites of the resin were more than the number of GA molecules, its adsorption capacity was higher in the single component adsorption model.

The pore structure of the resin might be altered to some degree after the adsorption of GA and GAMG, which, in turn, reduced the adsorption capacity of GA in the three-component adsorption model. In this regard, Temkin and the D-R isotherm model was more fit to describe the adsorption isotherm data. I observed that the D-R model was better than the Temkin model for characterizing the adsorption of GL, while the Terkin model was more capable of describing the adsorption isotherm of GAMG, which further confirmed the difference in the effects of GL and GAMG on the adsorption of GA. Previous research found that NKA-9 resin was the most appropriate for the separation of GAMG and GL, and the adsorption data fit the best to the Freundlich isotherm equation. The finding disagreed with my study partly because of the different properties between the two reins [32]. Additionally, it was found that the Terkin isotherm fit better to the adsorption of GA. The molecular size of GL and GAMG were so large that they blocked the diffusion of GA from aqueous solution to the pores of the resin as GL and GAMG coexisted with GA, thus leading to lessened GA adsorption [33]. The competitive adsorption between GL, GAMG, and GA could not be ignored. As displayed in Figure 2, the adsorption capacity for GA in the three-component system was reduced. This was essentially because both GL and GAMG were adsorbed preferentially in the mesopores and macropores. which were more accessible to these two adsorbates. As a result, the entrance of the micropores and smaller mesopores was partially blocked, making the adsorption sites less available for GA [33].

### 2.2. Static Desorption

#### The Effect of Different Eluents on the Desorption Ratio for GA, GAMG, and GL

**Figure 3:**
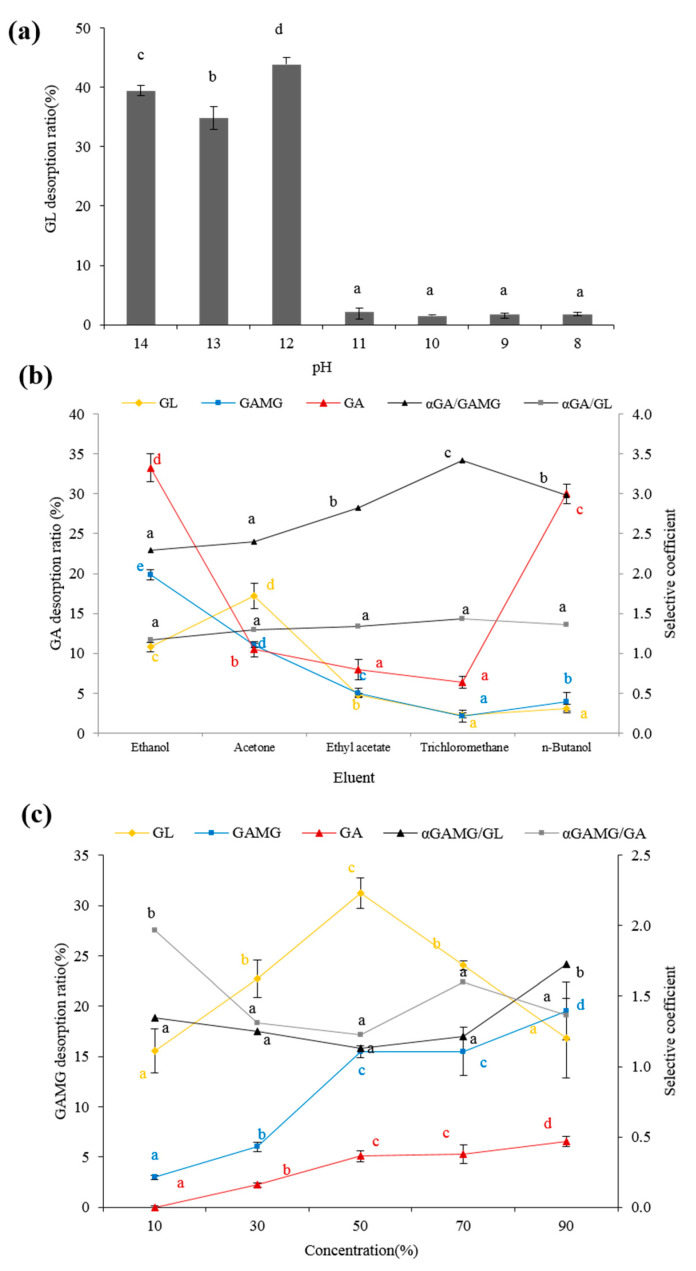
The effect of different eluents on desorption of GL, GAMG, and GA; (**a**) the effect of eluent pH value on desorption ratio of GL; (**b**) the effect of different organic solvents on desorption ratio of GA; (**c**) the effect of ethanol concentration on desorption ratio of GAMG. The letters a, b, c, d present the significance. The different letters indicated that was significant (*p* < 0.05), just the same letter indicated that was not significant (*p* > 0.05).

Figure 3a displays the effect of the pH of the distilled water on the desorption ratio. It was found that the desorption ratio for GL was very sensitive to the pH value, and a strong alkali solution resulted in a higher desorption ratio. GL is a weak acid with three carboxyl and five hydroxyl groups, and its dissociation constants are pKa_1_ = 2.76 ± 0.70, pKa_2_ = 2.81 ± 0.70, and pKa_3_ = 4.71 ± 0.70 [34]. When the pH is higher than 6 (above the pKa_3_ of GL), GL is deprotonated, and it behaves like a hydrophilic molecule (the corresponding salt form); thus, GL could easily dissolve in the aqueous phase for desorption. As a result, GL was desorbed with an aqueous solution at pH 12.

The elution process was that the partial adsorbate dissolved into the eluent and competed with the adsorbate for adsorption [28]. GA showed a weaker polarity; hence, the organic solvents were selected to elute GA. It was observed that the desorption ratio of GA with *n*-butanol was higher than that with other eluents, accompanied by a relatively high selective coefficient (α) (Figure 3b). Therefore, *n*-butanol was selected as an appropriate eluent.

The desorption ratio of GAMG increased with the elevation of the ethanol concentration. Desorption resulted from a dynamic balance between the dissolution into the eluent and the inter-molecular forces of the adsorption on the resin. Therefore, GAMG would be desorbed from the resin into the ethanol when the inter-molecular forces were less. At an ethanol concentration of 90%, the desorption ratio reached a maximum. The higher the concentration, the higher the selectivity coefficient αGAMG/GA was. Therefore, 90% ethanol was chosen for the dynamic desorption of GAMG.

### 2.3. The Dynamic Adsorption and Desorption

#### 2.3.1. The Dynamic Adsorption Curve

**Figure 4:**
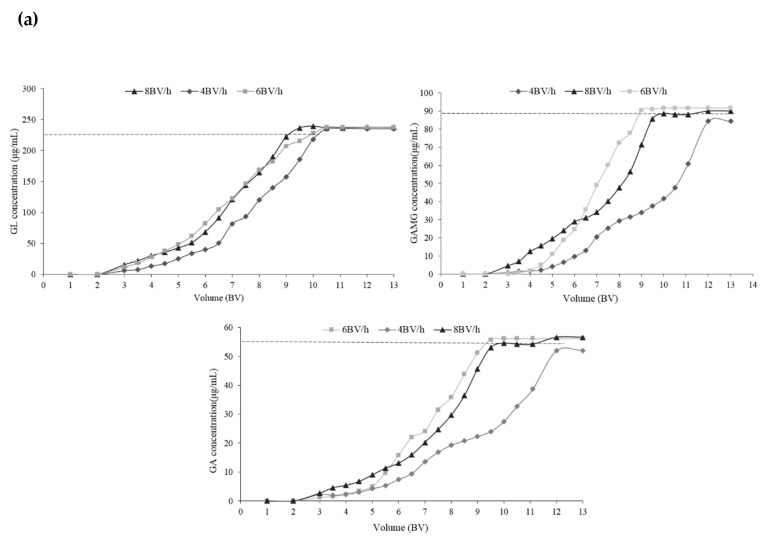

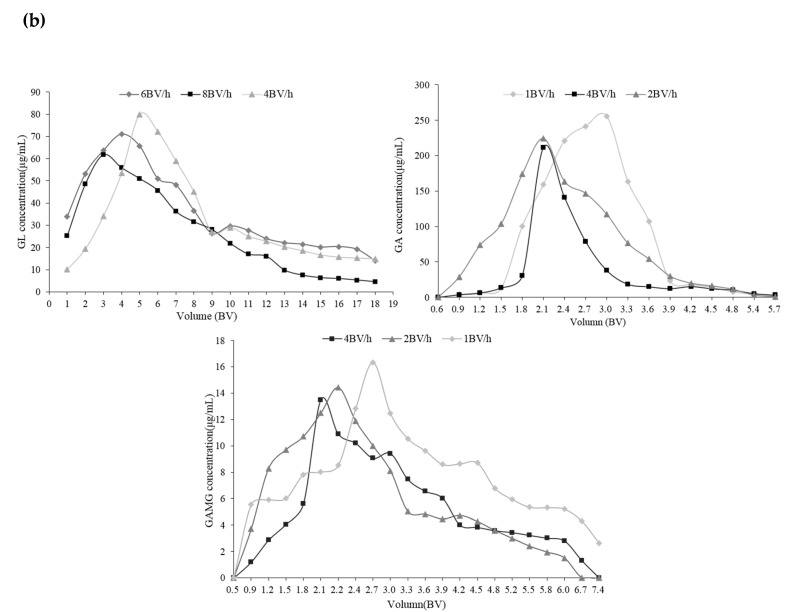
The desorption properties of GL, GAMG, and GA. (**a**) Dynamic breakthrough curves; (**b**) the desorption curves at different flow rates.

The dynamic adsorption properties of GL, GAMG, and GA on EXA50 are depicted in Figure 3a. According to the breakthrough curve of GL (Figure 4a), the adsorption breakthrough point for GL (*C*/*C*_0_ = 0.05) was 3.8 BV (bed volume, i.e., the volume of the resin) at a flow rate of 4 BV/h, 3 BV at 6 BV/h, and 2.8 BV at 8 BV/h. The adsorption breakthrough points for GAMG and GA were 5 BV at a flow rate of 4 BV/h, 4.5 BV at 6 BV/h, 3 and 3.2 BV at 8 BV/h, respectively. There was little absorbate in the effluent before 5 BV, because they were almost totally adsorbed onto the resin. With a rise from 5 BV to 10 BV, the adsorbate concentration in the effluent slightly increased, and then reached the adsorption saturation point at *C*/*C*_0_ = 0. After the saturation point, the concentrations in the effluent were close to those in the sample. The adsorption flow rate of 8 BV/h was too fast for the adsorbate to be adsorbed adequately on the resin. After considering both the adsorption capacity and the efficiency, 6 BV/h was preferred.

#### 2.3.2. Optimization of the Separation Condition

To optimize the dynamic desorption process, flow rates of 4, 6, and 8 BV/h were tested in the desorption process for GL (Figure 4b). The results showed that the flow rate was negatively associated with the desorption of GL. The slower the flow rate, the higher the concentration of the GL in the effluent. Therefore, 4 BV/h was preferred to desorb GL.

To determine the effect of the flow rate on the separation efficiency of GAMG and GA, flow rates of 1, 2, and 4 BV/h were tested. At the flow rate of 1 BV/h, three compounds were completely separated and the amount of GA was higher than with other higher flow rates. When the flow rate increased to 2 and 4 BV/h, the amount of GA reduced, and it was eluted together with GL and GAMG. According to the aforementioned results, the flow rate should be controlled at 1 BV/h in the desorption process of GA.

When the flow rate was 4 BV/h, the desorption processes of the three compounds were similar, making it difficult to separate them. In addition, the flow rate of 1 BV/h was undesirable, as low efficiency would limit the application. When the flow rate was increased to 2 BV/h, GAMG was separated from GA, while GAMG partially eluted with GL. Given that they are commonly mixed together as an edulcorator, 2 BV/h was selected.

#### 2.3.3. The Multi-Stage Desorption

**Figure 5:**
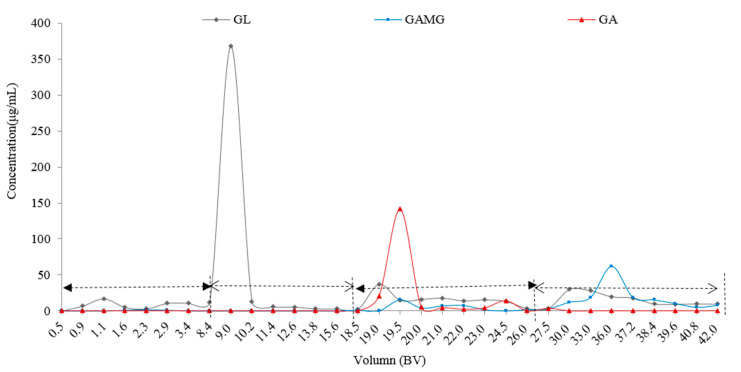
The multi-stage desorption curves of GA, GAMG, and GA.

Dynamic adsorption was performed at the flow rate of 6 BV/h. After reaching an adsorptive equilibration, the adsorbate-laden column was gradually eluted with water, *n*-butanol, and 90% ethanol, respectively. As shown in Figure 5, the elution between 8 BV and 15 BV with the aqueous solution of pH 12 gave a GL-rich fraction. From 15 BV to 26 BV, GA was collected, and GAMG appeared mainly after 30 BV, which was collected as a final product. As determined by HPLC, the purities of GL, GAMG, and GA were 90%, 65%, and 70%, respectively, which were first separated with the resin. The different purities depended on the separation method and selected reins. The purity of GAMG on NKA-9 resin was 85.02% [32], and purified GL on HPD-400 rein after removing the licorice flavonoids showed a purity of 88.95% [35].

## 3. Materials and Methods

### 3.1. Materials

Standards of GL (≥98%) and GA (≥98%) were purchased from Winherb Medical Science Company, Ltd. (Shanghai, China). GAMG (≥80%) was provided by Chun Li (Bioengineering Lab in Beijing Institute of Technology, Beijing, China). All reagents (HPLC-grade) were obtained from Merck Company (Shanghai, China). Other chemicals and solvents (analytical-grade) were purchased from Beijing Chemical Company (Beijing, China). EXA50 (surface area of 1000 m^2^/g, average pore diameter of 114 Å, non-polar) was purchased from Mitsubishi Chem. Co. (Tokyo, Japan).

### 3.2. Preparation of GL Derivants

GAMG and GA were produced in a subcritical water extraction apparatus (Model CWYF-2, Haihua Petroleum Research Instrument Company, Nantong, Jiangshu, China) as previously described by Fan et al. (2016). The hydrolysis was carried out at a constant pressure of 7.0 MPa. The GL solution (4 g dissolved in 100 mL 30% (*v*/*v*) ethanol solution) was purged at a constant flow rate (20 mL/min) via a liquid infusion pump (Model P6000, Beijing Chuangxin Tongheng Science and Technology Company, Beijing, China) into a sealed reaction vessel. The hydrolysis in a subcritical fluid was not only dependent upon the temperature, but also on the exposure time. The reaction parameters were optimized in Fan’s previous report, which included a temperature of 160 °C for 16 min [9]. At the end of the reaction, the hydrolysate was centrifuged and dried.

### 3.3. Static Adsorption and Desorption

In the preliminary experiments, the macroporous adsorption resin EXA50 (Residion, Mitsubishi Chem. Co., Tokyo, Japan) was selected to separate GL, GAMG, and GA (Figure S1 and Table S1 in the Supplementary Materials).

#### 3.3.1. Static Adsorption

Briefly, 0.2 g of resin was accurately weighed and mixed with 20 mL adsorption solution containing GL, GAMG, and GA with their initial concentrations of 0.4 mg/mL, 0.07 mg/mL, and 0.06 mg/mL, respectively. The mixture was shaken at 100 rpm/min for equilibration in a water bath at 25 °C, and then they were filtered and collected for analysis. The calculation equations of the equilibration adsorption capacities, *Qe* (mg/g) and adsorption rate, *A* (%) are as follows:

(1) $$Q_{e}\;=\;\left(C_{0}-C_{e}\right)\times \frac{V}{W}$$

(2) $$A\;=\;\frac{C_{0}-C_{e}}{C_{0}}\times 100\%$$

where, *C*_0_ (μg/mL) and *C_e_* (μg/mL) represent the adsorbate concentrations at the initial and at the equilibration stages, respectively. *V* (mL) is the volume of the adsorption solution and *W* (g) is the resin mass.

To optimize the appropriate adsorbate concentration and adsorbent dosage, the effect of the different initial concentration of GA at 0.06, 0.4, 0.8, 1.33, 1.44 mg/mL with constant concentrations of GL (0.4 mg/mL) and GAMG (0.07 mg/mL) (the data of the different initial concentrations of GAMG and GL are not shown in this study) were analyzed. The effects of the EXA50 resin mass (0.025, 0.05, 0.10, 0.15, 0.20, 0.25, and 0.30 g) on the adsorption efficiency were also discussed.

To obtain a better understanding of the adsorption process, the pseudo-first-order, pseudo-second-order, intra-particle diffusion kinetic model, Boyd’s model, Elonvich model, and Bangham model were used to estimate the adsorption kinetics of GL and its derivants. The best fit model was preferred based on the normalized standard deviation (Δ*Q*), accuracy factor (A*f*), and the correlation coefficient (*R*^2^) [36]. The A*f* value, used to evaluate the deviation between the calculated and fact values, was close to 1 to represents a better fit model [37]. The calculation equations were as follows:

(3) ∆Q(%) = 100×∑i = 1n[(Qt,exp−Qt,cal)Qt,exp]i2n−1

(4) $$A\;=\;\frac{C_{0}-C_{e}}{C_{0}}\times 100\%$$

where, *n* represents the number of data points, and *Q_t,exp_* and *Q_t,cal_* (mg/g) represent the experimental and kinetics-calculated sorption capacities at any time *t* (h), respectively.

(1) The pseudo first-order and pseudo second-order kinetics

The pseudo first- and second-order kinetic equation was proposed by Ho and McKay (1999), who hypothesized that the biosorption was followed by a second-order chemisorption and then proposed the pseudo first- and second-order kinetic equation [22]. Their linear forms are provided in Equations (5) and (6):

(5) $$\ln\left(Q_{e,1}-Q_{t}\right)\;=\;\ln Q_{t}-k_{1}\times t$$

(6) $$\frac{t}{Q_{t}}\;=\;\frac{1}{k_{2}Q_{e,2}^{2}}+\frac{t}{Q_{e,2}}$$

where *Q_e_* (mg/g) and *Q_t_* (mg/g) are the adsorption amount of GL (or GAMG or GA) at equilibrium and at any time *t*(h), respectively. *k*_1_ and *k*_2_ represent the adsorption rate constants of the pseudo-first order and the pseudo-second order, respectively.

(2) The intra-particle diffusion model

The intra-particle diffusion model assumes that the diffusion of the compounds is the rate-controlling step during the adsorption [38]. The model is given as the following equation:

(7) $$Q_{t}=k_{id}t^{\frac{1}{2}}+C$$

where, *k_id_* refers to the intra-particle diffusion rate constant (mg/(g·h^1/2^)), and *C* (mg/g) is the thickness of the boundary layer where a big value indicates a strong adsorption capacity.

(3) Boyd’s diffusivity model (BDM)

To consider the adsorption as a chemical phenomenon, Boyd’s kinetic model was proposed for the diffusion as carried out through the boundary liquid film [18]. The simplified form of the rate equation can be expressed as:

(8) $$\mathrm{In}\left[\frac{1}{1\;-{\;F}^{2}\left(t\right)}\right]=\;\frac{\pi^{2}D_{e}t}{R_{\alpha }^{2}}$$

where F(*t*) = *Q_t_*/*Q_e_* is the fractional attainment of the equilibrium at time *t* (h), and *De*(m^2^/s) and *R_α_* (m) represent the rate constant and the spherical adsorbent particle radius, respectively.

(4) Elonvich model

On the basis of the chemisorption phenomena, the Elovich kinetic model, a multilayer adsorption, assumes that the adsorption sites increase exponentially with ongoing adsorption [39]. The Elovich equation can be written as follows:

(9) $$Q_{t}\;=\;\frac{1}{\beta }\mathrm{In}\left(\alpha \beta \right)\;+\;\frac{1}{\beta }\mathrm{In}t$$

where *α* (mg/g·min) and *β* (g/mg) refer to the initial sorption rate and the desorption constant, respectively, which are correspondingly related to the extent of the surface coverage and activation energy during the chemisorption.

(5) Bangham model

The Bangham model assumes that the diffusion into the pores of the resin is the rate-controlling step [40]. The model equation is expressed as the following equation:

(10) $$\mathrm{loglog}\left(\frac{C_{0}}{C_{0}-Q_{t}\times m}\right)\;=\;\log\left(\frac{k_{0}\times m}{2.303V}\right)+\alpha \log t$$

where *C*_0_ (mg/L) is the adsorbate initial concentration, *V* (mL) is the solution volume, *m* (g/L) is the resin mass, *Q_t_* (mg/g) is the adsorption capacity at time *t* (h), and *α* (<1) and *k*_0_ (mL/g·L) are the Bangham constants.

#### 3.3.2. Adsorption Isotherm Modeling

Adsorption isotherms describe the equilibrium distribution of an adsorbate between the adsorbent and the liquid phase. To better understand the adsorption properties, different models were adopted. To evaluate the predictive ability of the above isotherm models, the root mean square error (RMSE) was used [41]:

(11) $$RMSE\;=\;\sqrt{\frac{\sum_{i\;=\;1}^{n}{\left(Q_{e,exp}-Q_{e,cal}\right)}_{i}^{2}}{n}}$$

where *Q_e,exp_* and *Q_e,cal_* (mg/g) are the experimental and isotherm model-calculated sorption capacities, respectively, and *n* is the number of data points.

(1) Langmuir isotherm

The Langmuir isotherm model considers that the adsorbent surface is covered with a monolayer of the adsorbate at specific homogeneous sites [42] and is represented as:

(12) $$Q_{e}\;=\;\frac{\alpha_{L}Q_{0}C_{e}}{1+\alpha_{L}C_{e}}$$

where *C_e_* (mg/L) means the initial adsorbate concentration at equilibrium, *Q_e_* (mg/g) is the amount adsorbed per unit weight of adsorbent; *Q*_0_ is the maximum adsorption capacity, and *a_L_* is the ratio between the adsorption and desorption. *K_L_* is defined as the solute absorptivity and is calculated as follows:

(13) $$K_{L\;}=\;Q_{0}\alpha_{L}$$

The characteristics of the Langmuir equation can be expressed by a dimensionless constant RL:

(14) $$R_{L}\;=\;\frac{1}{1+\alpha_{L}C_{0}}$$

where *C*_0_ (mg/L) represents the highest value of the adsorbate initial concentration. The *R_L_* value indicates whether the shape of the isotherm is unfavorable (*R_L_* > 1), linear (*R_L_* = 1), favorable (0 < *R_L_* < 1), and irreversible (*R_L_* = 0) [24].

(2) Freundlich isotherm

The Freundlich isotherm considers that the sorption process is non-ideal and reversible, which describes that the multilayer adsorption of the adsorbate onto the heterogeneous adsorbent surfaces was a non-uniform distribution with the heat during adsorption [41], and the model equation is written as follows:

(15) $$\ln Q_{e}\;=\;(\frac{1}{n})\ln C_{e}+\ln K_{F}$$

where *Q_e_* (mg/g) is the sorption capacity of a radionuclide at equilibrium, *K_F_* means the Freundlich constant, and *n* represents the heterogeneity factor.

(3) Temkin isotherm

Considering the chemisorption, the model assumes that the heat of adsorption of all the molecules in the layer decreases linearly with the coverage due to adsorbate-adsorbent interactions [41]. The equation and its linearized form are represented as follows:

(16) $$Q_{e}\;=\;B\ln K_{T}+B\ln C_{e}$$

(17) $$B\;=\;\frac{RT}{b_{T}}$$

where *b_T_* (J/mol) is a constant, and *K_T_* (L/mg) is the equilibrium binding constant.

(4) Dubinin-Radushkevich isotherm

Assuming that the adsorption curves are related to the porosity of the adsorbents, Dubinin proposed Dubinin-Radushkevich isotherm as follows [43]:

(18) $$\ln Q_{e}\;=\;-\beta \varepsilon^{2}+\ln Q_{0}$$

where, *β* (mol^2^/kJ^2^) is a isotherm constant related to the adsorption energy, and *ε* (kJ/mol),the adsorption potential, is correlated as follows:

(19) $$\varepsilon \;=\;R\cdot T\ln(1+\frac{1}{C_{e}})$$

where *T* means temperature (K), and *R* is the gas constant (8.314 × 10^−3^ kJ/mol·K).

#### 3.3.3. Static Desorption

Considering the similar physicochemical properties of GL, GAMG, and GA, multi-stage desorption tests were adopted. In terms of the different characteristics in polarity among them, the polarity order appeared as GL > GAMG > GA, therefore, the multi-stage desorption was performed as follows: after the adsorptive equilibration, the adsorbate-laden resin was initially washed by water solutions with different pH values (pH 8–14), then eluted by different organic reagents (anhydrous ethanol, ethyl acetate, *n*-butanol, acetone, and chloroform), and finally eluted by different concentrations of ethanol–water solutions (10%, 30%, 50%, 70%, and 90%) successively. After each desorption, the solutions were filtered and GL, GAMG, and GA were determined using HPLC. The adsorption capacity, *Q_e_* (mg/g), was calculated according to the following equation.

(20) $$Q_{d}\;=\;C_{d}\times \frac{V_{d}}{W}$$

(21) $$D_{r}\;=\;C_{d}\times \frac{V_{d}}{\left(C_{0}-C_{e}\right)\times V}\times 100\%$$

where *C*_0_ (μg/mL) and *C_e_* (μg/mL) represent the concentration of the adsorbent at the initial and equilibrium stage in adsorption, respectively; *C_d_* (μg/mL) represents the adsorbate concentration in the eluent. *V_d_* (mL) and *V* (mL) indicate the volumes of the eluent and adsorption solution, respectively, and *W* (g) is the resin mass.

To optimize the desorption selectivity for the targeted compound against the other two compounds, the selectivity coefficient *α* was defined as Equation (22).

(22) αGAGAMG = KGAKGAMG

where *K* is the distribution ratios of different compounds. *K* (L/g) was calculated using Equation (23) [44].

(23) $$K\;=\;\frac{Q_{d}}{C_{d}}$$

### 3.4. Dynamic Adsorption and Desorption

#### 3.4.1. Fixed-Bed Column Adsorption

Dynamic adsorption and desorption trials were performed in a glass column (1.5 cm × 15 cm) packed with EXA50 resin. Briefly, 3.5 g of the resin was densely packed in a glass column. The adsorption solution was passed through the resin column at flow rates of 4, 6, and 8 BV/h, and the concentrations of GL, GAMG, and GA in the effluent from the column were continuously recorded until they reached the initial concentration.

#### 3.4.2. Optimization of the Desorption Conditions for GL, GAMG, and GA

After the breakthrough run of adsorption, the resin column was eluted with different eluents for the different compounds using multi-stage desorption, and different flow rates (1, 2, and 4 BV/h) for each desorption process were investigated. The concentrations of GL, GAMG, and GA in the effluent were recorded until they were close to zero.

### 3.5. Statistical Analysis

All the experiments and measurements were performed in triplicate. Analysis of variance (ANOVA) was performed using SPSS 18.0.

## 4. Conclusions

In the present study, macroporous resin was successfully applied to isolate GL and its derivants from the hydrolysis product of GL in subcritical water. The adsorption and desorption properties were investigated systematically. It was concluded that the kinetic data were fit best to the pseudo-second-order kinetics model and that isotherms could be appropriately described by the Temkin, D-R, and Freundlich isotherm models. GL, GAMG, and GA were significantly enriched in the effluent. As a result, this method proved to be an effective way to separate GL and its derivants because of its advantages including simplicity, low cost, high purification efficiency, and ease of scale-up. This indicates the potential for the large-scale purification and preparation of GL and its derivants and the feasible preparation of sweeteners from licorice in the future.

## Figures and Tables

**Table 1:** The adsorption kinetic parameters of GL, GAMG, and GA onto EXA50 resin.

The Kinetic Parameters for Adsorption of GL, GAMG, and GA						
Model	Parameters					
**Pseudo first-order**	***k***_**1**_	***Q_e,cal_*(mg/g)**	***Q_e,exp_*(mg/g)**	***R*^2^**	***Q*(%)**	***Af***
GL	0.066	8.661	10.888	0.815	18.05	1.458
GAMG	0.122	2.948	2.638	0.890	15.89	1.182
GA	0.116	3.081	2.468	0.845	19.05	1.294
**Pseudo second-order**	***k*_2_**	***Q_e,cal_*(mg/g)**	***Q_e,exp_*(mg/g)**	***R*^2^**	***Q*(%)**	***Af***
GL	0.069	10.225	10.888	0.999	7.20	1.042
GAMG	0.113	2.880	2.638	0.986	10.68	1.089
GA	0.094	2.744	2.468	0.980	9.25	1.104
**Intra-particle diffusion**	***K_id_*[g/(g·h^1/2^)]**	***C***		***R*^2^**	***Q*(%)**	***Af***
GL	1.147	4.892		0.862	7.012	1.0587
GAMG	0.446	0.663		0.810	14.38	1.1083
GA	0.450	0.430		0.857	10.518	1.0896
**Boyd’s diffusion**	***De*(m^2^/s)**			***R*^2^**	***Q*(%)**	***Af***
GL	7.7 × 10^−6^			0.838	16.068	1.1685
GAMG	1.1 × 10^−5^			0.901	11.758	1.1035
GA	9.4 × 10^−6^			0.847	13.051	1.1026
**Elovich diffusion**	***α*[mg/(g·min)]**	***β*(g/mg)**		***R*^2^**	***Q*(%)**	***Af***
GL	0.724	0.642		0.940	4.54	1.037
GAMG	0.037	1.677		0.890	12.76	1.097
GA	0.027	1.707		0.925	11.02	1.090
**Bangham diffusion**	***α***	***k*_0_((mL/(g·L))**		***R*^2^**	***Q*(%)**	***Af***
GL	0.260	0.053		0.936	4.61	1.039
GAMG	0.460	0.016		0.886	11.47	1.097
GA	0.541	0.013		0.943	8.45	1.067

∆*Q*, normalized standard deviation; A*f*, accuracy factor; *R*^2^, correlation coefficient; *Q_t,exp_*, *Q_t,cal_* (mg/g), the experimental and kinetics-calculated sorption capacities at any time *t*(h); *k*_1_, *k*_2_, the adsorption rate constants of the pseudo-first-order and the pseudo-second-order; *De*(m^2^/s), the rate constant; *k_id_* (mg/(g·h^1/2^)), the intra-particle diffusion rate constant; *α* (mg/(g·min)), initial sorption rate; *β* (g/mg), desorption constant; *α* and *k*_0_(mL/(g·L)), the Bangham constants.

**Table 2:** The isotherm parameters of GA obtained from single GA solutions.

Model	Parameters					
**Langmuir**	***Q*_0_ (mg/g)**	***a_L_* (L/mg)**	***R_L_***	***K_L_* (L/g)**	***R*^2^**	**RMSE**
	34.722	0.009	0.068	0.328	0.988	1.104
**Freundlich**	**1/*n***	***K_F_* (mg/g)**			***R*^2^**	**RMSE**
	0.719	0.482			0.993	0.974
**Temkin**	***b_T_* (J/mol)**	***K_T_* (L/mg)**	**−∆*G* (kJ/mol)**		***R*^2^**	**RMSE**
	7.809	0.165	4.384		0.842	1.303
**D-R**	***Q*_0_ (mg/g)**	***ε (*kJ/mol)**	***β* (10^−5^, (mol^2^/J^2^)**		***R*^2^**	**RMSE**
	25.977	0.224	2.000		0.888	1.121

RMSE, root mean square error; *a_L_*, the ratio between the adsorption and desorption; *K_L_*, the solute adsorptivity; *Q*_0_, the maximum adsorption capacity; *R_L_*, dimensionless constant; *K_F_*, the Freundlich constant; *n*, the heterogeneity factor; *b_T_*, the heat of adsorption; *K_T_*, the equilibrium binding constant.

**Table 3:** The isotherm parameters obtained from three-component solutions.

Model	Adsorbate	Parameters					
**Langmuir**		***Q*_0_ (mg/g)**	***a_L_* (L/mg)**	***K_L_* (L/g)**	***R_L_***	***R*^2^**	**RMSE**
	GL	13.175	0.573	7.550	0.005	0.853	1.223
	GAMG	3.211	1.843	5.919	0.009	0.859	1.567
	GA	2.276	2.871	6.534	0.008	0.889	1.078
**Freundlich**		**1/*n***	***K_F_* (mg/g)**			***R*^2^**	**RMSE**
	GL	0.4162	0.956			0.824	2.012
	GAMG	0.471	0.384			0.882	1.712
	GA	0.505	0.281			0.922	0.989
**Temkin**		***b_T_* (J/mol)**	***K_T_* (L/mg)**	**−∆*G* (KJ/mol)**		***R*^2^**	**RMSE**
	GL	3.004	0.092	5.812		0.829	2.145
	GAMG	0.749	0.453	1.930		0.903	1.567
	GA	0.567	0.509	1.643		0.937	0.898
**D-R**		***Q*_0_ (mg/g)**	***β* (10^−5^, mol^2^/J^2^)**	**ε (KJ/mol)**		***R*^2^**	**RMSE**
	GL	9.456	50.000	0.032		0.854	1.898
	GAMG	2.173	1.000	0.224		0.880	1.679
	GA	1.498	0.070	0.267		0.824	2.123

RMSE, root mean square error; *a_L_*, the ratio between the adsorption and desorption; *K_L_*, the solute adsorptivity; *Q*_0_, the maximum adsorption capacity; *R_L_*, dimensionless constant; *K_F_*, the Freundlich constant; *n*, the heterogeneity factor; *b_T_*,the heat of adsorption; *K_T_*, the equilibrium binding constant.

## Supplementary Materials

The following are available online. Figure S1: The structures of GL, GAMG and GA. Figure S2: The effects of the initial concentration of GA and adsorbent dosage on the static adsorption efficiency. Figure S3: The isothermals model of GL, GAMG and GA. Table S1: Adsorption capability and desorption rate of different resins.
