# Supplementary material for: Separation of Glycyrrhizic Acid and Its Derivants from Hydrolyzation in Subcritical Water by Macroporous Resin
Source: Molecules. 2020 Sep 19;25(18):4305. doi: 10.3390/molecules25184305 (PMC7570570; doi:10.3390/molecules25184305)
Supplement: Supplementary file 1 [file molecules-25-04305-s001.pdf]

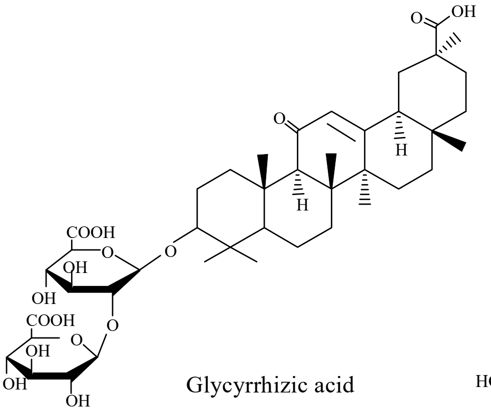

Glycyrrhizic acid

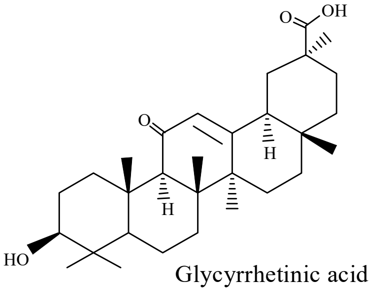

Glycyrrhetinic acid

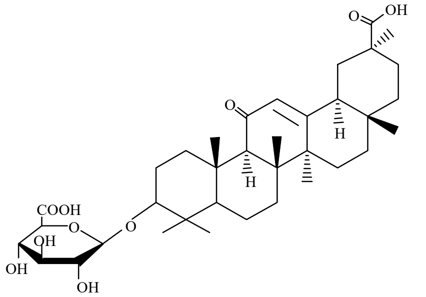

Glycyrrhetinic acid 3-O-mono-β-D-glucuronide

Fig. S1 The structures of GL, GAMG and GA.

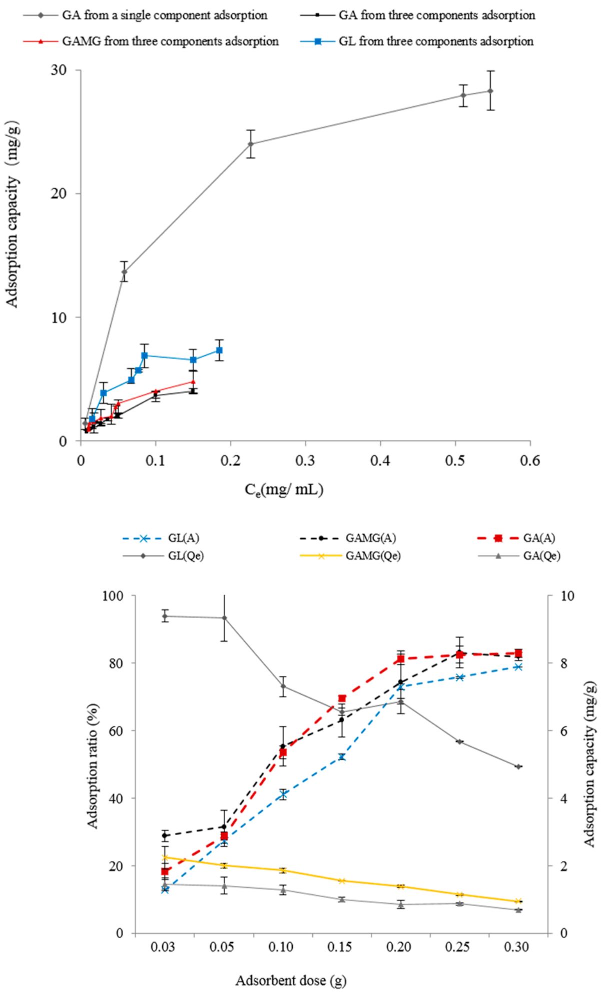

Fig. S2 The effects of the initial concentration of GA and adsorbent dosage on the static adsorption efficiency;

1S. The effect of GA initial concentration on the adsorption efficiency

The initial concentration of adsorbate was a critical parameter in the adsorption process, the influence of GA initial concentration on the adsorption efficiency was evaluated in present study (Fig.S2). It could be found that the adsorption capacity increased sharply with the rise of GA concentration and then kept a gradually increase. This phenomenon might be interpreted by the fact that the concentration gradient at high GA concentration solution induced higher driving force for mass transfer (Ding et al., 2012), and then the slight increase might be due to the adsorption saturation.

2S. The effect of adsorbent dosage on the adsorption efficiency

The effect of varying dosage of the adsorbent was investigated, the adsorption capacity and adsorption ratio as the function of resin dosage are presented in Fig. S2. It showed an increase in the adsorption ratio (A) of GL, GAMG and GA with the rise in dosage of the resin up to certain limit and then the increase became negligible. In addition, the adsorption capacity (Q_e) was decreased with the increase in the dosage of the resin. The increase in the adsorption with raising dosage of the resin was likely due to the rise in adsorbent surface area and availability of more adsorption sites (Barkakati et al., 2010). Therefore, the dosage of 0.2 g EXA50 resin was selected for further adsorption experiments .

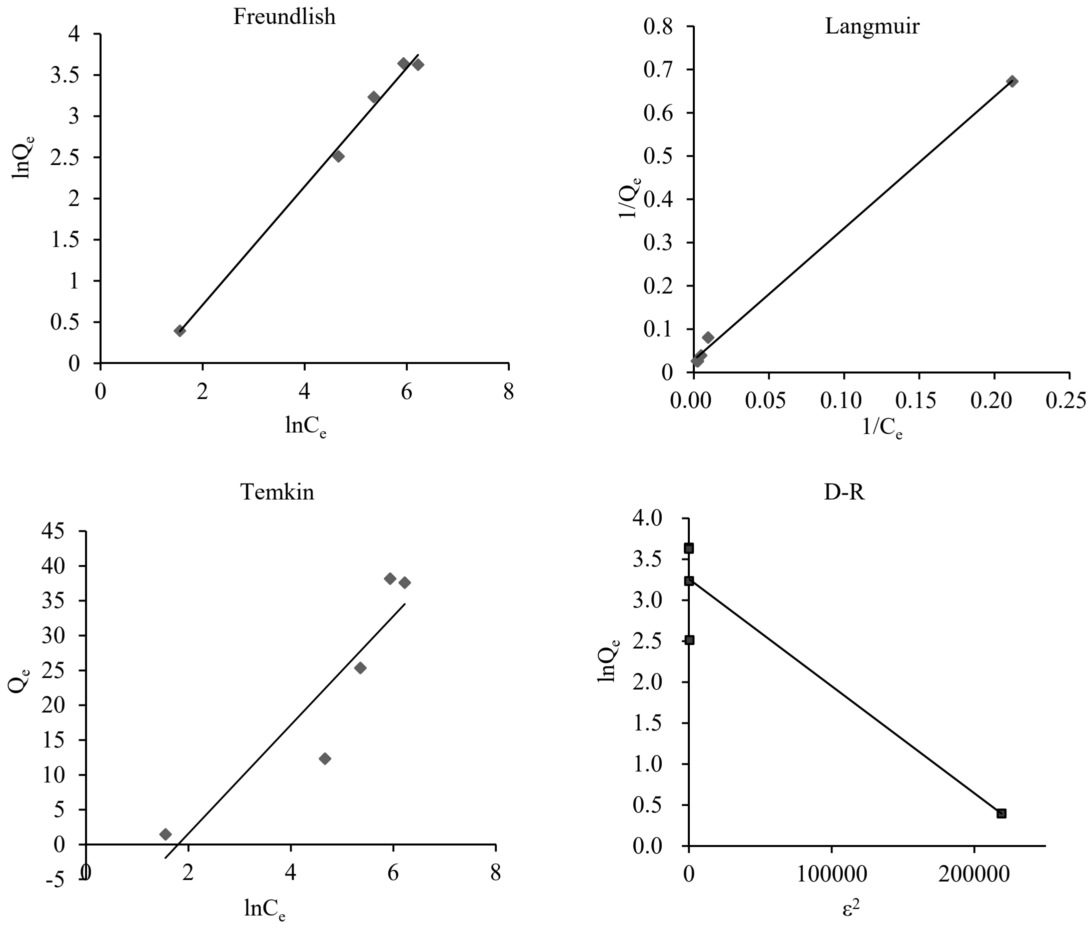

Fig. S3 The isothermals model of GL, GAMG and GA.

Table S1 Adsorption capability and desorption rate of different resins

Resin	GL		GAMG		GA	
	Adsorption capacity (mg/g)	Elution rate (%)	Adsorption capacity (mg/g)	Elution rate (%)	Adsorption capacity (mg/g)	Elution rate (%)
XDA4	15.72±0.8^{d}	74.09±6.0$^{b'}$	5.12±0.2^{k}	19.95±5.6$^{i'j'}$	4.30±0.2^{qr}	5.44±0.8$^{p'q'r'}$
HZ803	8.70±1.2^{b}	91.51±2.3$^{f'g'}$	3.57±0.4^{i}	31.83±8.8$^{l'm'}$	3.06±0.3^{p}	7.54±1.3$^{r's'}$
HP700	16.92±2.0^{de}	78.74±5.5$^{b'c'd'}$	5.47±0.2^{kl}	32.36±6.0$^{m'}$	4.31±0.1^{qr}	8.20±0.9$^{s'}$
SP70	18.68±0.8^{fg}	59.74±3.6$^{a'}$	5.71±0.3^{lmn}	19.01±3.5$^{i'}$	4.38±0.2^{r}	8.26±1.6$^{s'}$
SP207	17.62±0.5^{ef}	83.36±5.9$^{b'c'd'}$	5.50±0.1^{kl}	28.68±3.5$^{k'l'm'}$	4.23±0.1^{qr}	5.42±0.3$^{p'q'r'}$
XDA1180	17.74±1.8^{ef}	76.51±8.6$^{b'c'}$	5.56±0.4^{klm}	31.97±4.4$^{l'm'}$	4.34±0.^{3r}	7.00±1.2$^{q'r's'}$
HP100	20.34±0.5^{g}	79.18±6.8$^{b'c'd'}$	6.00±0.1^{mn}	25.98±5.9$^{k'l'}$	4.68±0.1^{s}	5.17±0.9$^{p'q'}$
XDA7HP	13.80±0.2^{c}	93.66±2.4$^{g'}$	5.69±0.1^{lmn}	44.13±0.7$^{n'}$	4.72±0.1^{t}	14.38±2.2$^{t'}$
HP2MGL	5.12±0.6^{a}	80.22±6.7$^{b'c'd'}$	4.07±01^{j}	53.14±1.0$^{o'}$	4.06±0.1^{q}	16.04±1.7$^{t'}$
EXA45	18.10±3.0^{ef}	82.80±4.7$^{c'd'e'}$	5.51±1.0^{kl}	25.18±6.8$^{j'k'}$	4.44±0.5^{rs}	4.59±0.3$^{p'}$
EXA50	22.17±0.5^{h}	85.54±2.7$^{d'e'f'}$	6.53±0.1^{o}	26.54±1.8$^{k'l'm'}$	4.75±0.1^{t}	8.29±1.2s'
SP207	19.79±1.0^{g}	89.06±1.2$^{e'f'g'}$	6.06±0.2^{n}	28.00±1.3$^{k'l'm'}$	4.75±0.1^{t}	4.48±0.6$^{p'}$
SP825	20.34±0.3^{g}	85.28±6.4$^{d'e'f'}$	6.07±0.1^{n}	25.32±1.5$^{j'k'}$	4.79±0.1^{t}	7.37±1.2$^{q'r's'}$
EXA118	20.25±0.2^{g}	83.00±7.5$^{c'd'e'}$	6.09±0.1^{n}	30.81±1.6k$^{'l'm'}$	4.76±0.1^{t}	5.66±0.2$^{p'q'r'}$

Note: The results were expressed as mean±standard deviation (n=3), with the different letters marked that was significant ($P < 0.05$).

References:

Ding L, Deng HP, Wu C, Han X(2012) Affecting factors,equilibrium, kinetics and thermodynamics of bromideremoval from aqueous solutions by MIEX resin. Chem Eng J 181: 360–370.

Barkakati P, Begum A, Das ML, Rao PG (2010)Adsorptive separation of Ginsenoside from aqueous solution by polymeric resins: Equilibrium, kinetic and thermodynamic studies. Chemical Engineering Journal, 161(1): 34-45.
